# Liquid Metal Microrobots for Magnetically Guided Transvascular Navigation

**DOI:** 10.1002/adma.202518382

**Published:** 2025-12-19

**Authors:** Xiaohui Ju, Roshan Velluvakandy, Xianghua Wu, Miguel Angel Merlos Rodrigo, Zbyněk Heger, Kamila Bendíčková, Jan Frič, Martin Pumera

**Affiliations:** ^1^ Future Energy and Innovation Laboratory Central European Institute of Technology Brno University of Technology Purkyňova 123 Brno 61200 Czech Republic; ^2^ State Key Laboratory of Advanced Technology for Materials Synthesis and Processing International School of Materials Science and Engineering Wuhan University of Technology 122 Luoshi Road Wuhan 430070 P.R. China; ^3^ Department of Chemistry and Biochemistry Mendel University in Brno Zemedelska 1665/1 Brno 61300 Czech Republic; ^4^ Center of Advanced Innovation Technologies Faculty of Materials Science and Technology VSB ‐ Technical University of Ostrava 17. listopadu 2172/15 Ostrava 70800 Czech Republic; ^5^ International Clinical Research Center St. Anne's University Hospital Pekařská 664/53 Brno 60200 Czech Republic; ^6^ International Clinical Research Center Faculty of Medicine Masaryk University Kamenice 753/5 Brno 62500 Czech Republic; ^7^ Advanced Nanorobots & Multiscale Robotics Laboratory Faculty of Electrical Engineering and Computer Science VSB ‐ Technical University of Ostrava 17. listopadu 2172/15 Ostrava 70800 Czech Republic; ^8^ Department of Medical Research China Medical University Hospital China Medical University No. 91 Hsueh‐Shih Road Taichung 40402 Taiwan; ^9^ Department of Chemical and Biomolecular Engineering Yonsei University 50 Yonsei‐ro, Seodaemun‐gu Seoul 03722 South Korea

**Keywords:** cross‐membrane, gallium‐indium, liquid metal, organ‐on‐a‐chip, radiopaque, reconfigurable, targeted delivery

## Abstract

Soft microrobots, compared with their rigid counterparts, offer superior adaptability in dynamic and confined biological environments. Here, magnetically‐guided liquid metal microrobots composed of gallium‐indium alloys embedded with Fe nanoparticles are introduced. The unique combination of magnetic maneuverability, high surface tension, intrinsic radiopacity, and deformability allows liquid metal‐based microbots to overcome limitations of both hard microrobots and fragile droplet‐based systems. Under magnetic actuation, liquid metal‐based magnetic microrobots exhibit controllable rolling and upstream locomotion resembling neutrophil‐like navigation, enabling precise maneuvering even against physiological flow. Bridging in vitro with in vivo experiments, quail egg chorioallantoic membrane models are used to demonstrate guided transport of these microrobots through blood vessels, accumulation at tumor xenografts, and migration within subcutaneous tissues. Moreover, their strong X‐ray visibility enables real‐time fluoroscopic tracking, validated in porcine heart vasculature. Importantly, liquid metal‐based magnetic microbots can cross endothelial barriers in a vascular flow‐on‐a‐chip platform, while maintaining endothelial biocompatibility. By integrating deformability, magnetic steerability, and imaging visibility, liquid metal‐based microrobots establish a powerful platform for minimally invasive transvascular navigation. This work highlights the potential of liquid metal‐based magnetic microrobots for targeted drug delivery, image‐guided therapy, and intelligent biomedical interventions.

## Graphical abstract

Liquid metal‐based microrobots combine magnetic steering, intrinsic X‐ray visibility and softness, to navigate blood vessels even against flow. Under clinically relevant magnetic fields, liquid metal microrobots roll along vessel walls, cross endothelial barriers, and accumulate in target tissues. Their robust motion and imaging contrast highlight their potential for minimally invasive, image‐guided biomedical interventions.

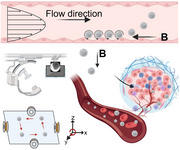

## 1 Introduction

Non‐invasive tools capable of navigating through complex biological environments are attracting increasing attention for advancing targeted diagnostics, precision therapy, and other intelligent biomedical interventions.^[^ ^1^ ^]^ Whether in the treatment of vascular diseases, localized drug delivery, or image‐guided surgery, the ability of the microdevices to actively explore and respond to dynamic physiological conditions remains a critical challenge.^[^ ^2^ ^]^ Conventional approaches often rely on passive agents or rigid instruments that lack the adaptability and control needed to access confined or obstructed regions deep within the body.^[^ ^3^ ^]^ To address these limitations, new adaptive strategies considering soft‐bodied materials are emerging to integrate active navigation, real‐time imaging, and mechanical compliance.^[^ ^4^ ^]^

Magnetically actuated microrobots offer a powerful platform for active navigation through biological systems.^[^ ^5^ ^]^ These tiny devices can be remotely guided through fluidic environments and can access regions that are otherwise unreachable by conventional methods. Recent studies have demonstrated promising intravascular and endovascular applications of magnetically guided microrobots for targeted navigation, thrombolysis, and vessel imaging, highlighting the translational potential of this technology.^[^ ^6^, ^7^, ^8^ ^]^ While magnetic microrobotic swarms have shown promise in lab settings, their rigid mechanical structures often struggle with the soft and dynamic nature of biological tissues.^[^ ^9^ ^]^ The mismatch in mechanical properties can lead to poor adaptability, limited deformation, and increased risk of tissue damage or loss of functionality in vivo.^[^ ^10^ ^]^

**Figure 1:**
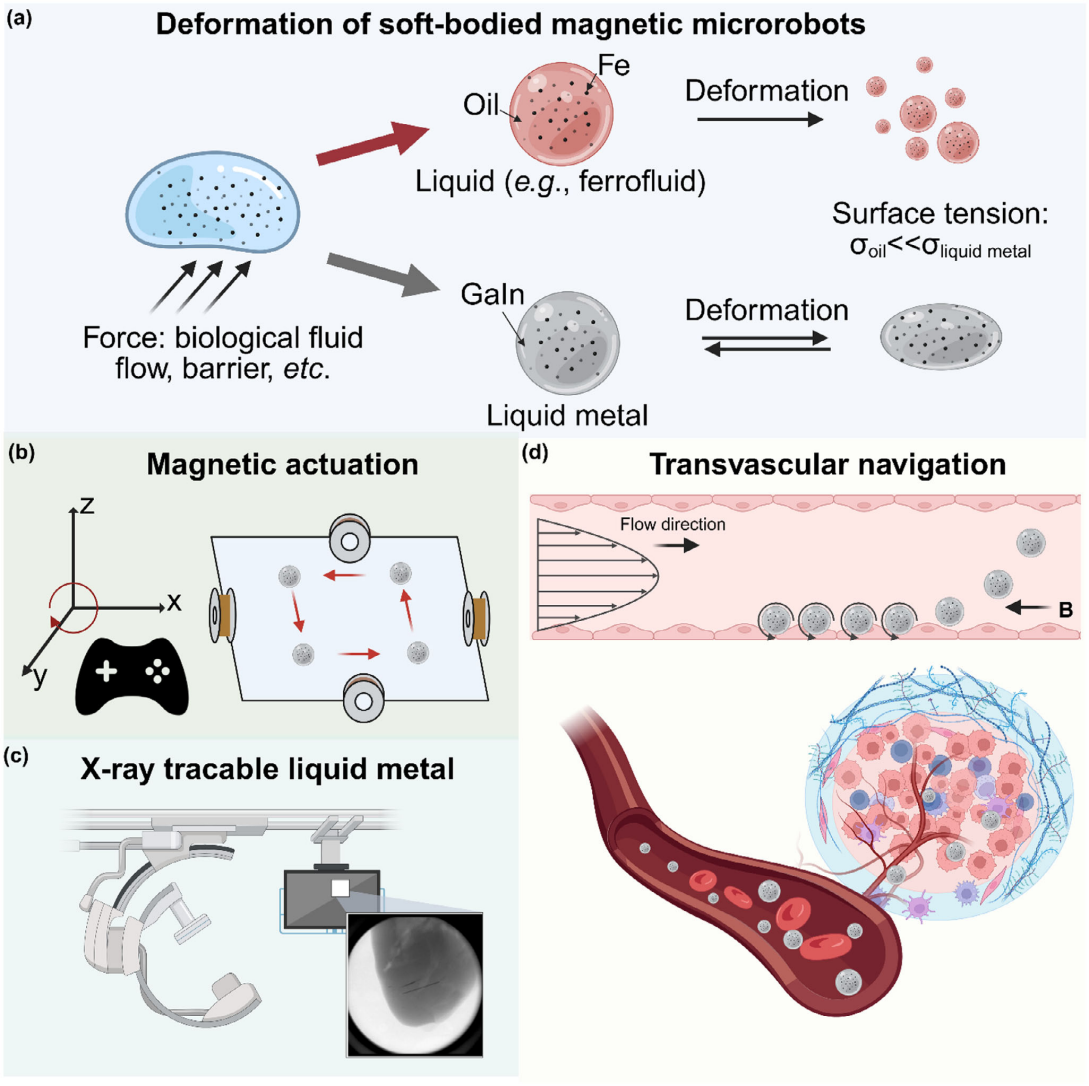
Deformable liquid metal magnetic microrobots (LMbots) for magnetically guided transvascular navigation. a) Deformation comparison between oil‐based and liquid metal‐based soft magnetic microrobots. b) Controlled navigation of LMbots through adjustment of the applied magnetic field. c) LMbots exhibit X‐ray radiopaque and traceable properties, enabling real‐time in vivo monitoring. d) Controlled transvascular navigation of LMbots enables targeting deep tissues against blood flow under magnetic actuation. B (with arrows) indicates the direction of the applied magnetic field.

Soft‐bodied microrobots, constructed by embedding magnetic particles into deformable materials, have been explored as a solution to this problem.^[^ ^11^ ^]^ By utilizing soft matrices such as elastomers or hydrogels, these systems achieve greater compliance and better adapt to biological structures.^[^ ^12^ ^]^ Yet, many of these materials face challenges in maintaining their shape and function under physiological flow conditions. Liquid‐based soft small‐scale robots, particularly those made from magnetic aqueous or oil‐based droplets, have been miniaturized and used for magnetic actuation and shape transformation.^[^ ^13^, ^14^ ^]^ However, their low surface tension and susceptibility to fragmentation under stress make them unreliable in fast‐flowing or high‐pressure biological environments.^[^ ^15^ ^]^ Liquid metal‐based alloys provide an optimal platform due to their fluidity at body temperature, near‐zero elastic modulus, and exceptionally high surface tension, which ensures shape stability even in turbulent biological flows.^[^ ^16^ ^]^ Unlike aqueous or oil‐based soft robots prone to fragmentation and instability due to low surface tension, liquid metal‐based microrobots remain cohesive and bioinert, minimizing undesired interactions in vivo (**Figure** **1**a).^[^ ^17^, ^18^ ^]^

Most microrobots designed for biomedical applications are introduced into the body through the bloodstream,^[^ ^1^ ^]^ but reaching their therapeutic targets often requires them to move beyond the vascular system and into surrounding tissues.^[^ ^17^ ^]^ This demands not only precise control over their navigation within blood vessels, but also the ability to actively interact with and cross the vascular barriers.^[^ ^19^ ^]^ Inspired by the natural immune response of neutrophils, which roll along the endothelium, adhere to inflamed sites, and transmigrate across vessel walls,^[^ ^20^ ^]^ we developed magnetically controlled microrobots based on gallium‐indium (GaIn) liquid metal that mimic this multistep behavior (Figure 1). Unlike neutrophils, which rely on biochemical signaling and receptor‐mediated adhesion, the liquid metal magnetic microrobots (LMbots) achieve analogous motion purely through physical interactions based on hydrodynamic effects and magnetic actuation rather than biological recognition processes. Our results show that these microrobots can navigate either along or against fluid flow conditions in a biological environment, cross narrow or obstructed vessels, and be reliably tracked in real time using X‐ray fluoroscopy thanks to their intrinsic radiopacity. The integration of magnetic actuation, mechanical adaptability, and active imaging capability positions this platform as a promising tool for minimally invasive transvascular exploration, with strong potential in targeted diagnostics, image‐guided therapy, targeted drug delivery, and intelligent biomedical interventions.

## 2 Results and Discussion

### 2.1 Fabrication of Liquid Metal Magnetic Microrobots

**Figure 2:**
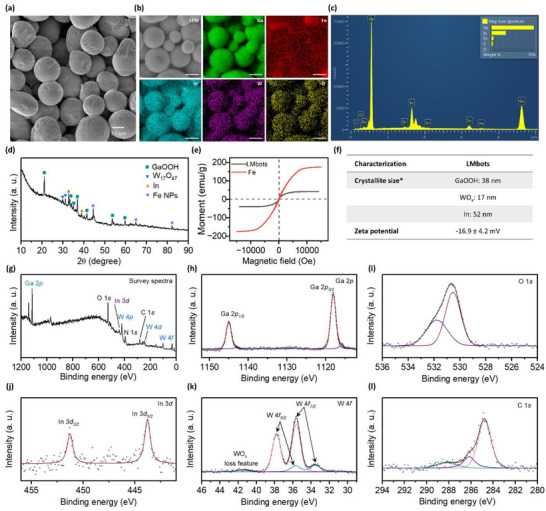
Physicochemical characterization of LMbots. a) SEM micrographs and b) EDX mapping of LMbots. Scale bar: 2.5 µm. c) EDX spectrum of LMbots. d) XRD patterns of LMbots. e) Magnetization curves of LMbots and Fe nanoparticles as reference. f) Crystalline sizes of different species and zeta potential. ^*^Analysis was calculated based on the assigned XRD pattern. XPS spectra of LMbots are presented from g–l), as (g) survey spectra; (h) Ga 2*p*; (i) O 1*s*, (j) In 3*d*; (k) W 4*f*; and (l) C 1*s*.

The LMbots were constructed by incorporating Fe nanoparticles into the liquid metal matrix containing a mixture of gallium (Ga) and indium (In). Indium has a melting point of 156.6 °C, while for Ga it is ≈29.76 °C. When mixed in a 3:1 ratio (Ga: In), the resulting alloy exhibits a significantly lower melting point than either of the pure metals, ranging from 15 to 20 °C, due to the eutectic behavior of the Ga–In binary system.^[^ ^17^, ^21^, ^22^ ^]^ After the surface oxide film of the liquid metal alloy was removed by acid treatment, vigorous shaking facilitated sufficient contact between Fe nanoparticles and the Ga–In alloy, allowing the Fe nanoparticles to be infiltrated and form GaIn–Fe microparticles.^[^ ^23^ ^]^ Sonication was carried out to break the liquid metal into smaller and homogenous microparticles. To prevent the undesired migration of solid Fe nanoparticles to the GaIn surface, sonication was performed in a sodium tungstate (Na_2_WO_4_) solution. The presence of tungstate ions (WO_4_^2−^), which possess a higher reduction potential than the Ga/Ga^3+^ redox couple, facilitates a galvanic reaction at the freshly exposed Ga surface during sonication. This galvanic interaction passivates the liquid metal surface and helps stabilize the Fe nanoparticles inside the liquid metal droplets, thereby suppressing their migration.^[^ ^24^ ^]^ The scanning electron microscopy (SEM) image in **Figure** **2**a shows the synthesized LMbots with smooth surfaces, excluding the possibility of surface decoration by Fe nanoparticles. Energy dispersive X‐ray spectroscopy (EDX) mapping revealed the signals for Ga, In, Fe, W, and O, and the asymmetrical spatial distribution of Fe signals confirms its heterogeneous distribution inside LMbots (Figure 2b). Weak signals of O and W indicated very thin layers (negligible W) of WO_x_ species coated on the surface of these LMbots (Figure 2c). The sizes of these LMbots ranged from 2 to 10 µm with a negative surface zeta potential due to the WO_x_ coatings. X‐ray diffraction (XRD) pattern of LMbots presented a mixed structure composed of oxidized Ga and W species, as shown in Figure 2d,f, as well as metallic In and Fe, corresponding to the previously reported structure of GaIn‐Fe microparticles.^[^ ^17^, ^22^ ^]^ The existence of Fe and its active magnetic properties were confirmed by vibrating sample magnetometry (VSM). Pure Fe nanoparticles exhibited a typical ferromagnetic hysteresis loop with a saturation magnetization intensity ≈170 emu g^−1^. After being incorporated into the GaIn alloys, the assembled LMbots displayed weaker ferromagnetism with intensity ≈26 emu g^−1^ (Figure 2e). Using the more surface‐sensitive characterization technique of X‐ray photoelectron spectroscopy (XPS), the chemical composition of the WO_x_‐coated GaIn‐Fe LMbots was examined in greater detail (Figure 2g–l). XPS has a surface penetration depth of less than 10 nm, making it particularly effective for probing surface features of microscale particles, where surface signals dominate over the bulk composition. As shown in Figure 2h, the predominant Ga species detected was oxidized gallium (≈95%), likely in the form of GaOOH, consistent with the crystalline structure identified by XRD. A minor fraction (≈5%) of metallic Ga was also detected within the probing depth. The O 1*s* spectrum (Figure 2i) exhibits a dominant peak at 530.5 eV, attributed to metal‐oxygen bonds (W─O and Ga─O), along with a higher binding energy shoulder at 531.8 eV, corresponding to O–H or adsorbed H_2_O species. Due to the limited penetration depth of XPS, the In 3*d* signal was relatively weak (Figure 2g,j), suggesting that In resided deeper within the core of LMbots. The W 4*f* core‐level spectrum (Figure 2k) indicates the presence of at least two oxidation states, W^2+^ and W^3+^, with the tungsten oxides primarily localized at the particle surface.

### 2.2 Magnetic Control of LMbots under Flow Conditions

**Figure 3:**
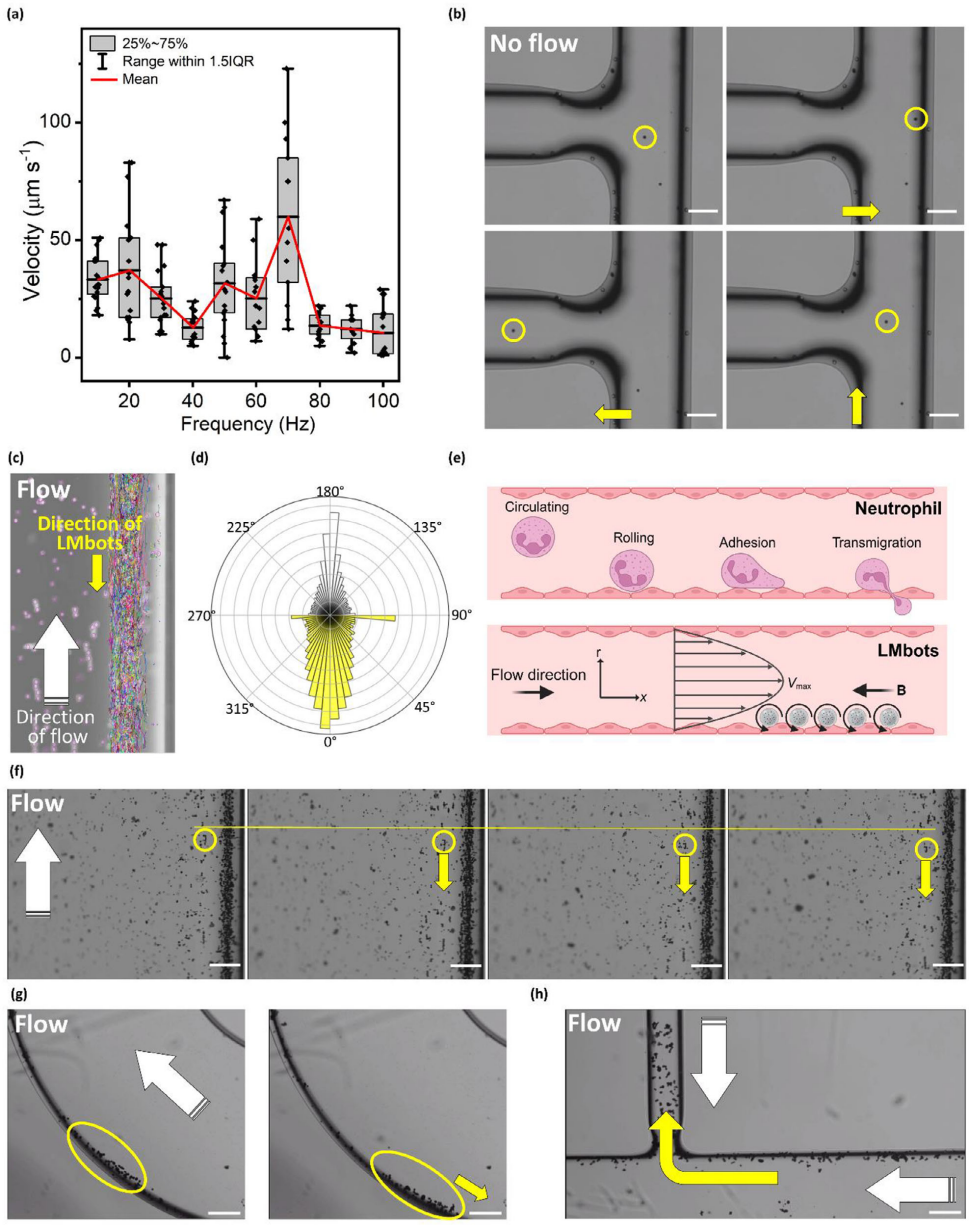
Magnetic steerability of LMbots enables neutrophil‐like tumbling behavior against flow. a) The velocity of LMbots movement was analyzed under a rotating magnetic field with different frequencies ranging from 10 to 100 Hz at 3 mT. b) Controlled steering of individual LMbots in various directions under actuation by a rotating magnetic field, in the absence of flow. c) LMbots exhibit rolling motion along channel walls against the flow at a flow rate of 40 cm min^−1^ in a microfluidic channel, resembling neutrophil‐like migration in the blood vessels. Individual particles (LMbots and tracer particles were tracked). d) Rose diagram based on individual particle analysis showing that tracer particles followed the flow direction, while LMbots moved in the opposite direction (180^°^) under magnetic field control. e) Scheme illustrating the process of neutrophil vascular immobilization and transvascular migration (upper panel), and the fluid flow velocity gradient where LMbots migrate toward the vessel walls and move upstream under the influence of a rotating magnetic field (lower panel). f) Tracking the position of individual LMbot clusters under a rotating magnetic field in the presence of flow. LMbots are going against the flow direction. The flow condition is the same as in b. g) Controlled steering of LMbots following curved turns against flow under actuation by a rotating magnetic field, with flow conditions at 40 cm min^−1^ in a microfluidic channel. h) Controlled steering of LMbots following branched turns against flow under actuation by a rotating magnetic field, with flow conditions at 40 cm min^−1^ in a microfluidic channel. All scale bars represent 100 µm unless otherwise indicated.

To demonstrate that LMbots can be magnetically driven in a real‐time manner, the velocity of LMbots at different frequencies was investigated by adjusting the parameters of the 3D electromagnetic rotating field. The correlation between the velocity and the magnetic field frequency is shown in **Figure** **3**a. The highest velocity of LMbots at 60 µm s^−1^ was achieved at a magnetic field strength of 3 mT and a frequency of 70 Hz, under which LMbots were moving in a rolling mode. Optimal performance is closely tied to the step‐out behavior of LMbots, which exhibit two distinct step‐out frequencies at 50 and 70 Hz, unlike solid magnetic particles that typically show only one. This dual behavior highlights the liquid‐solid hybrid nature of LMbots. In solid magnetic systems, step‐out occurs when magnetic torque can no longer synchronize the magnetic moment with the external field. For LMbots, varying the frequency affects the alignment of Fe nanoparticle chains within the liquid metal matrix. At high fluid velocities, the magnetic attraction becomes insufficient to overcome viscous drag, causing chain breakage. This leads to a sharp loss in torque, reflected in a sudden drop in velocity and a cliff‐like decrease in rotational speed.^[^ ^17^ ^]^ To prevent undesired magnetic dipole‐dipole aggregation during collective operation, the LMbots exploit both surface and dynamic magnetic stabilization. The presence of a negatively charged WO_x_/GaOOH surface layer imparts electrostatic and steric repulsion, which minimizes close contact under actuation. Moreover, the use of a continuously rotating magnetic field, rather than a static one, dynamically averages magnetic dipole interactions and prevents head‐to‐tail chaining. Operating at higher rotation frequencies (>50 Hz) further suppresses transient clustering by promoting rapid reorientation of magnetic moments. Together, these surface and field‐based effects enable stable, dispersion‐free swarm behavior of LMbots under flow.

Controllable locomotion of LMbots was observed via the optical microscope under the rotating magnetic field. The navigation was achieved by adjusting the direction of the magnetic field. LMbots were introduced into a microfluidic channel with a width of ≈200 µm to simulate the confined spaces of human blood vessels. Their navigability was first tested in a condition without fluid flow. As shown in Figure 3b, individual LMbots can immediately respond to the changes in the magnetic field, achieving the designed direction control. They can move in a straight path forward or in the opposite direction backward when the polarity of the magnetic field is altered. In addition, LMbots demonstrated L‐shape turns in the channel as shown in Video S1 (Supporting Information). These controllable navigation capabilities are particularly important for biomedical applications, where microrobots must cross confined and restricted environments with high precision to reach specific target sites.

An additional critical factor in navigating microrobots within biological environments is the influence of biofluid dynamics, such as blood flow within blood vessels. The practical design of microrobots must therefore account for and overcome the effects of these fluidic forces to reach their intended destinations effectively. To demonstrate this capability, we further evaluated the maneuverability of the microrobots in microfluidic channels under flow conditions. The tests were conducted at a flow velocity of 40 cm min^−1^ (6.7 mm s^−1^) in a microfluidic channel, which approximates the blood flow speed typically observed in small arterioles or venules.^[^ ^25^ ^]^ As shown in Figure 3c and Video S2 (Supporting Information), when a magnetic field was applied opposite to the direction of fluid flow (flow direction is indicated by the tracer particles), LMbots migrated toward the channel wall and rolled along it while simultaneously moving against the flow, rather than moving against the flow in the center of the tube. Single‐particle tracking analysis, presented in the Rose plot of Figure 3d, further confirms this opposing motion between LMbots and the flow direction. This behavior arises from the wall effect in fluid dynamics, as illustrated in Figure 3e. This effect is driven by the reduced flow velocity near the vessel walls, where there is a velocity gradient near the boundary of the solid‐liquid interface. Video S2 (Supporting Information) visualizes this near‐wall behavior, showing that LMbots in the intermediate region can roll or move slightly against the flow at 6.7 mm s^−1^, while at 10 mm s^−1^ the rolling becomes markedly slower and the LMbots remain nearly stationary, confirming the flow‐dependent stability predicted by the critical velocity analysis. The stable rolling behavior of the LMbots near the vessel wall results from a balance between magnetic, viscous, and frictional forces. A simplified analysis shows that the critical flow velocity for stable adhesion is proportional to the magnetic field strength, susceptibility, particle size, and wall friction, and inversely proportional to the fluid viscosity (Note S1, Supporting Information). Under a bulk flow of 10 mm s^−1^, the local near‐wall velocity decreases to ≈0.4 mm s^−1^ due to the no‐slip boundary, which can be counterbalanced by the magnetic‐adhesive force at 3 mT and field gradients ≈100 T m^−1^. This explains the experimentally observed region‐dependent behavior, where LMbots near the wall maintain adhesion or roll slowly against the flow, while those in the channel center are swept downstream.

In biological systems, such wall‐directed behavior is observed in sperm navigating along reproductive tract walls or bacteria like *E. coli* swimming near surfaces. Notably, the motion of LMbots closely resembles that of neutrophils, which migrate toward vessel walls, rolling along the endothelium before they adhere,^[^ ^26^ ^]^ which are key steps in their immune surveillance and response (Figure 3e). Figure 3f shows that some LMbots remained in the close‐to‐central flow region, farther from the channel wall, and these particles moved significantly more slowly than those located closer to the wall. While the rolling motion of LMbots along the channel wall visually resembles that of neutrophils, it arises purely from hydrodynamic wall effects rather than from biochemical adhesion and signaling. Here, the term “neutrophil‐like” is therefore used only as a physical analogy to describe the wall‐guided rolling and upstream migration pattern, not to imply a shared biological mechanism. The neutrophil‐inspired behavior of the microrobots along the wall for upstream fluid flow has been reported previously by Ahmed et al.,^[^ ^27^ ^]^ where the designed system utilized a magnetic field to induce particle self‐assembled aggregates, and these aggregates migrate toward the channel wall due to the induced radiation force of the acoustic field. Unlike previous reports, LMbots presented here exhibited spontaneous swarming toward the channel wall, driven solely by the fluid velocity gradient. In this region of minimized flow velocity, the reduced drag force allowed the magnetic field to more effectively induce upstream movement of LMbots. This effect was further validated in curved channels (Figure 3g) and branched channels with flow (Figure 3h). By programming the direction of the magnetic field, LMbots were guided not only to move upstream in straight channels but also to navigate through U‐shaped curves (Video S3, Supporting Information) and enter branched regions (Video S4, Supporting Information) where the flow direction was reversed. It is important to note that, unlike the navigating microrobots in extremely fast blood flow found in the aorta and its major branches, where vessel diameters typically range from 2 to 3 cm,^[^ ^28^, ^29^ ^]^ the system tested here is more representative of smaller blood vessels, such as arterioles and venules, which have diameters in the range of 100 to 300 micrometers. This makes the approach more applicable to microvascular environments.

The synthesized LMbots exhibited moderate variation in size and morphology (typically 2 to 10 µm in diameter), which arises from the intrinsic fluidic nature of GaIn alloys and the breakup dynamics during ultrasonic dispersion. Such nonuniformity is characteristic of liquid‐metal‐based microrobots and can cause minor differences in the induced magnetic moment, viscous drag, and interfacial tension, thereby influencing actuation dynamics under magnetic fields. Larger LMbots tend to experience stronger magnetic torque, while smaller ones show slightly lower step‐out frequencies, which may result in mild trajectory dispersion during collective motion. Nevertheless, this variation does not significantly affect overall magnetic responsiveness, as propulsion is mainly governed by the embedded Fe nanoparticle distribution and total magnetic torque rather than exact geometry. Similar particle‐size‐dependent magnetic responses have been reported for related systems.^[^ ^17^, ^21^, ^22^ ^]^ Repeated magnetic washing was applied to narrow the particle size distribution, and the liquid nature of LMbots allows local deformation and internal flow adjustment that further mitigate shape‐dependent differences during actuation.

### 2.3 Targeted Navigation of LMbots in Chorioallantoic Membrane Model

The chorioallantoic membrane (CAM) model is a well‐established, cost‐effective alternative to mammalian models for studying angiogenesis, tumor growth, and drug delivery.^[^ ^30^ ^]^ As a highly vascularized extraembryonic membrane that mimics the complex vasculature of organs like the liver, spleen, and lungs, the CAM offers a high‐throughput, in vivo‐like environment for direct observation of angiogenesis, tumor progression, and nanoparticle interactions under physiologically relevant conditions.^[^ ^31^ ^]^ Traditionally performed using chicken embryos, the CAM assay has also been adapted to quail (*Coturnix japonica*), which provides advantages such as shorter developmental cycles, smaller egg size, lower maintenance costs, and increased experimental throughput.^[^ ^32^ ^]^

**Figure 4:**
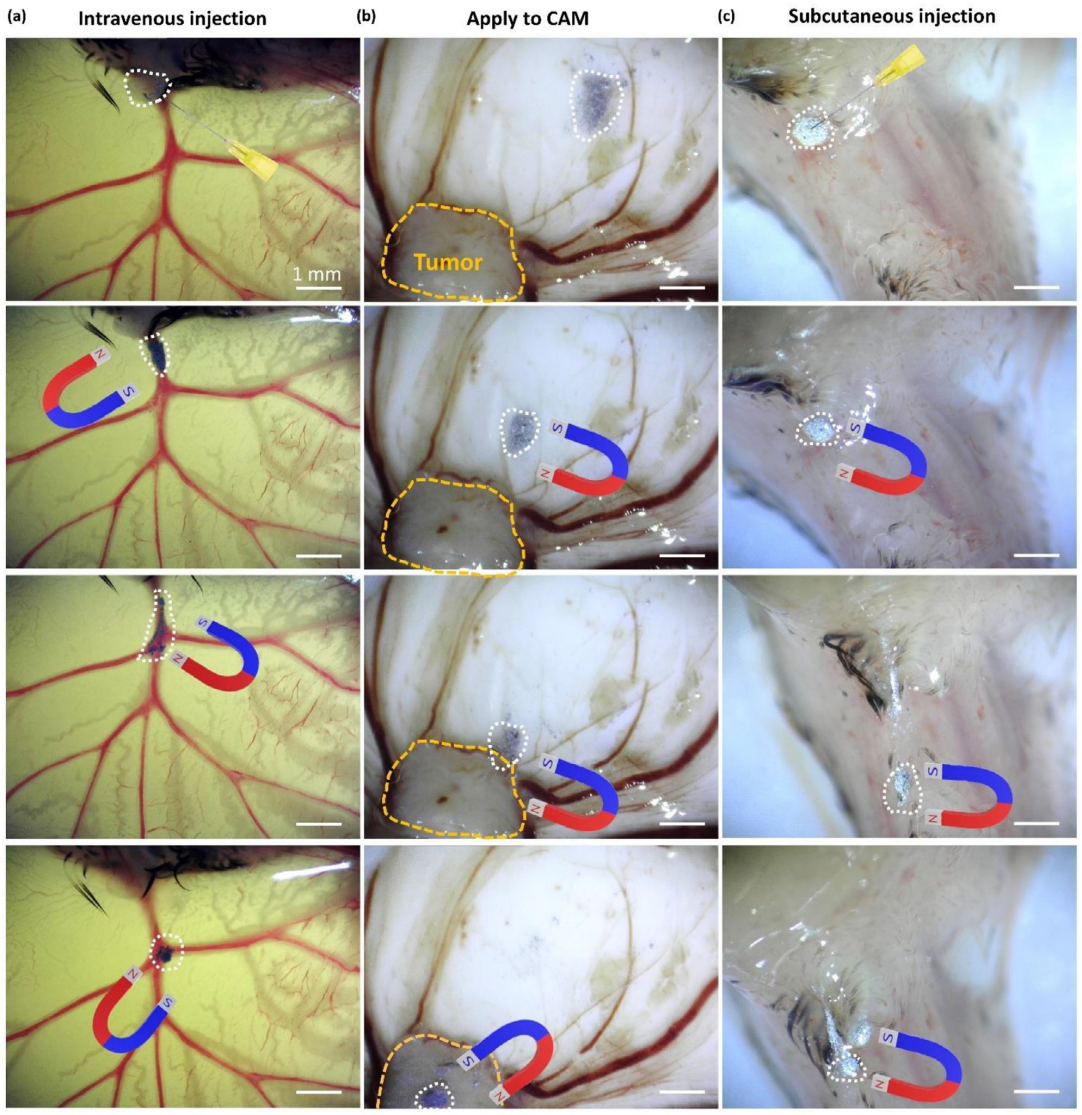
LMBots navigation in ex ovo quail embryos under magnetic actuation. a) Directed movement of LMbots following intravenous injection into a major blood vessel via a permanent magnet. b) Targeted migration of LMbots applied onto the CAM toward a human prostate tumor xenograft via a permanent magnet. c) Subcutaneous injection of LMbots into the neck region of quail chick embryos, followed by magnetic field‐guided movement through the surrounding tissue.

To investigate LMbots’ behavior within vascular networks, we conducted a series of experiments to evaluate LMbots’ maneuverability in the ex ovo biological environment. First, the navigation of LMbots was tested in the vasculature network with blood flow. LMbots were injected into a major blood vessel and exposed to an external magnetic field (**Figure** **4**a). Before magnetic actuation, LMbots exhibited passive diffusion along the direction of blood flow. Upon magnetic stimulation, LMbots demonstrated coordinated movement and accumulated along the vessel, confirming magnetically guided transport within the vasculature. To further evaluate the potential of LMbots for targeted drug delivery, we guided these particles toward a human prostate tumor xenografted on CAM, using the external magnetic field (Figure 4b). The application of an external magnetic field enabled the controlled movement and localized accumulation of LMbots directly at the site of the human prostate tumor, without observable complications. This approach highlights the potential to concentrate on therapeutic agents via LMbots at targeted tumor sites while minimizing off‐target effects on surrounding tissues. To explore their subcutaneous navigational capabilities, we further injected LMbots beneath the neck skin of quail embryos and guided them using an external magnetic field (Figure 4c; Video S5, Supporting Information). LMbots exhibited coordinated motion along the subcutaneous tissue, demonstrating their potential for noninvasive therapeutic delivery beneath the skin.

LMbots demonstrate precise magnetic maneuverability in biologically relevant models. Guided by external magnetic fields, they can target blood vessels, tumors, and subcutaneous tissues within quail CAM systems. Their successful navigation against blood flow, through branched vasculature, and via subcutaneous injection marks a significant step in targeted microrobotics. These results highlight their ability to operate in dynamic, physiologically challenging environments. As a noninvasive and adaptable platform, LMbots hold strong potential for precision medicine when combined with an advanced magnetic navigation system available in most clinical settings (such as MRI‐guided systems or custom electromagnetic coil arrays), enabling localized therapy with reduced side effects. Their effectiveness across angiogenic, tumor, and skin models underscores their promise for next‐generation biomedical interventions.

### 2.4 X‐Ray Contrast Properties of LMbots Ex Vivo

Accurate visualization of microrobots using clinically available imaging techniques is essential for precise control and navigation within complex anatomical vessel networks. Effective tracking enables safe and targeted biomedical interventions, enhancing the reliability and functionality of microrobotic applications in clinical settings. Radiopaque materials are therefore critical for enhancing the visibility of microrobots under X‐ray fluoroscopy and computed tomography (CT), widely available and routinely used imaging modalities in clinical environments. Developing microrobots with sufficient radiopacity ensures compatibility with existing medical infrastructure and supports real‐time monitoring during in vivo procedures. Furthermore, most contrast agents are liquid solutions with densities close to that of water, resulting in limited radiopacity and passive diffusion within the bloodstream. This lack of directional control prevents targeted delivery of contrast agents to specific sites, highlighting the need for microrobots composed of radiopaque materials that can be actively guided and precisely localized under X‐ray fluoroscopy for more effective and controllable biomedical interventions.

**Figure 5:**
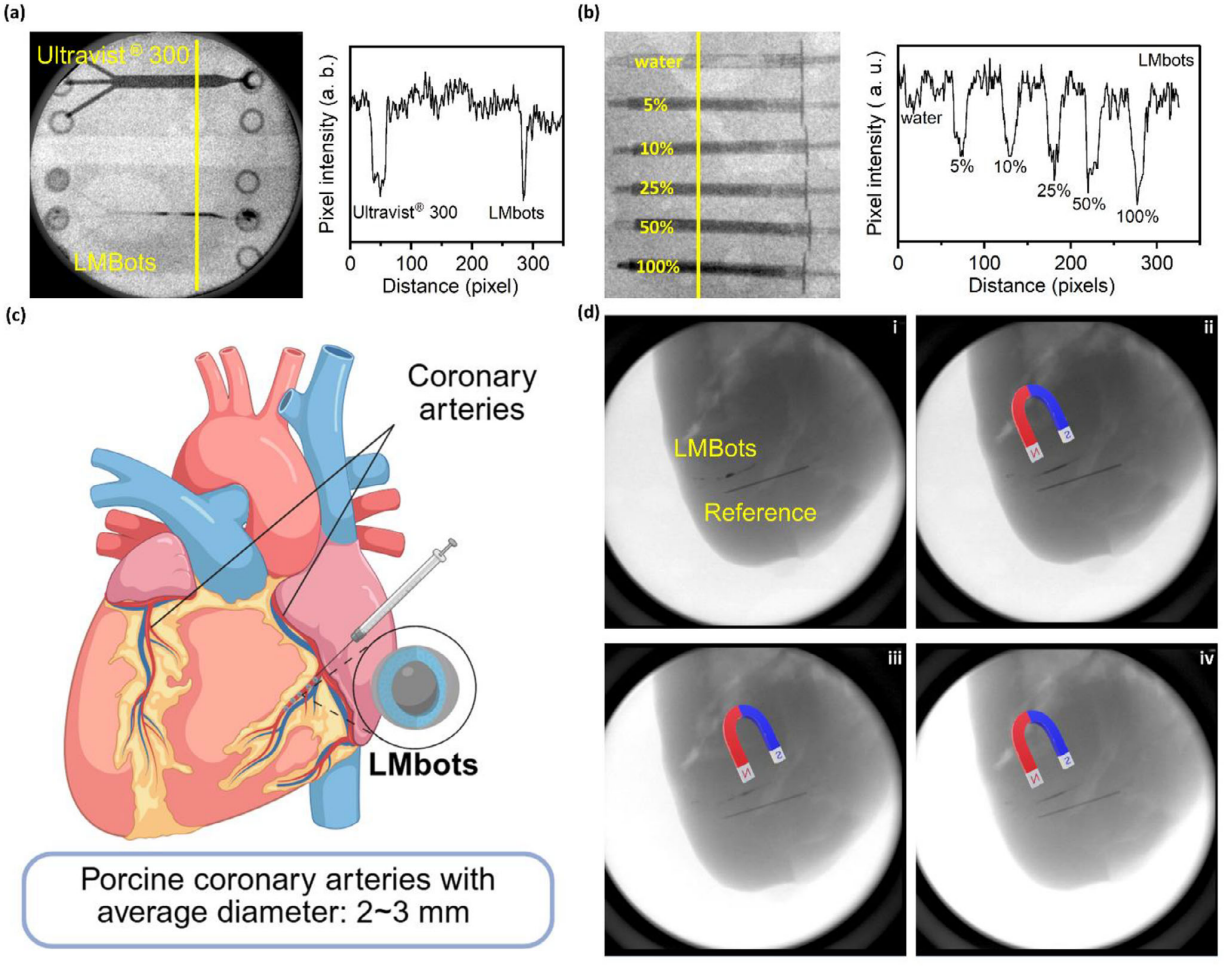
Evaluation of the ability of LMbots to act as active X‐ray contrast agents. a) X‐ray fluoroscopy image comparing a microfluidic channel filled with commercial iodine‐based contrast agent (Ultravist^®^ 300) and GaIn‐based LMbots, along with corresponding pixel intensity analysis. b) Quantitative analysis showing that X‐ray attenuation, represented by pixel intensity, increases with higher concentrations of LMbots. c) Schematic illustration of LMbots injection into the coronary arteries of an ex vivo porcine heart for contrast imaging. d) X‐ray image of LMbots infused into the coronary artery, with a metal needle inserted into the myocardium as a radiographic reference. LMbots were guided by an external magnetic field and distributed smoothly along the artery wall. (i) to (iv) show time‐lapse sequential images of LMbots being navigated along the coronary vasculature by the applied magnetic force.

Here, we examined the radiopacity of room‐temperature GaIn‐based LMbots as potential imaging contrast agents for X‐ray‐based angiography. The X‐ray fluoroscopy image shown in **Figure** **5**a compares the contrast effect of LMbots and commercial iodine‐based contrast agent Ultravist^®^ 300 (300 mg of I mL^−1^). GaIn‐based LMbots produced a superior contrast effect and enabled the visualization of their confined aggregation in the channel without flow. Unlike Ultravist^®^, which tends to diffuse rapidly and lose spatial precision, LMbots remained localized, forming well‐defined, high‐contrast clusters. This property is particularly advantageous in static or low‐flow environments, such as occluded vessels or during targeted therapy. To quantify the contrast effect, pixel intensity analysis of the X‐ray images was conducted. Figure 5b shows that the radiographic attenuation, measured in grayscale intensity, increases with the concentration of LMbots, indicating a dose‐dependent enhancement in radiopacity. At clinically relevant concentrations, the contrast values of LMbots were found to be comparable to those of Ultravist^®^, affirming their potential as effective radiopaque agents. These findings suggest that LMbots could either complement or replace traditional agents in specific applications, especially when prolonged imaging or localized retention is desired.

To extend these findings to more tissue‐relevant conditions, LMbots were infused into the coronary arteries of porcine hearts ex vivo (Figure 5c). A second metal needle was inserted into the myocardium as a radiographic reference. As shown in Figure 5d, under X‐ray fluoroscopy, the infused LMbots maintained strong radiopacity, clearly outlining the arterial structures. This demonstrates that LMbots are not only effective in synthetic channels but also exhibit consistent performance in complex biological tissues, where tissue density and scattering could otherwise diminish contrast visibility. Importantly, one of the unique advantages of LMbots is their magnetic responsiveness. When exposed to an external magnetic field generated by a permanent magnet, LMbots were actively navigated along the coronary vasculature (Figure 5d from i to iv). This was achieved despite the lack of physiological blood flow in the ex vivo setting. Throughout this magnetically guided movement, LMbots remained visible under X‐ray fluoroscopy.

Previous research has shown that liquid gallium can serve as a potent contrast agent when infused into the arteries of porcine hearts and kidneys, significantly enhancing vessel visibility by several orders of magnitude compared to conventional contrast agents.^[^ ^33^ ^]^ The GaIn alloy, known for its chemical stability, does not react with water or biological fluids and has been demonstrated to be safe for various biomedical applications, especially when contrasted with the toxic liquid metal mercury.^[^ ^34^ ^]^ The combination of strong radiopacity and the ability to be actively guided makes LMbots a highly promising platform for both diagnostic imaging and minimally invasive therapeutic interventions.

### 2.5 Deformation and Traversing Behaviors of LMbots Under Static Conditions

Recent intravascular microrobotic systems have shown the feasibility of magnetically guided navigation and targeted intervention inside blood vessels. For instance, Wang et al. demonstrated swarm microrobots capable of traversing branched microvessels under magnetic control for thrombus removal and vascular repair,^[^ ^6^ ^]^ and the same group also reported steerable microrobots achieving precise motion in physiological flow conditions.^[^ ^7^ ^]^ LMbots with magnetic maneuverability offer another key advantage for biomedical applications: their softness and deformability. Unlike soft‐bodied microrobots made from elastomers or hydrogels, which often struggle to maintain their shape under physiological conditions, LMbots retain structural integrity. Similarly, pure liquid‐based soft materials are prone to fragmentation under stress, limiting their use. In contrast, liquid metal‐based soft microrobots achieve a balance between softness and high surface tension.

**Figure 6:**
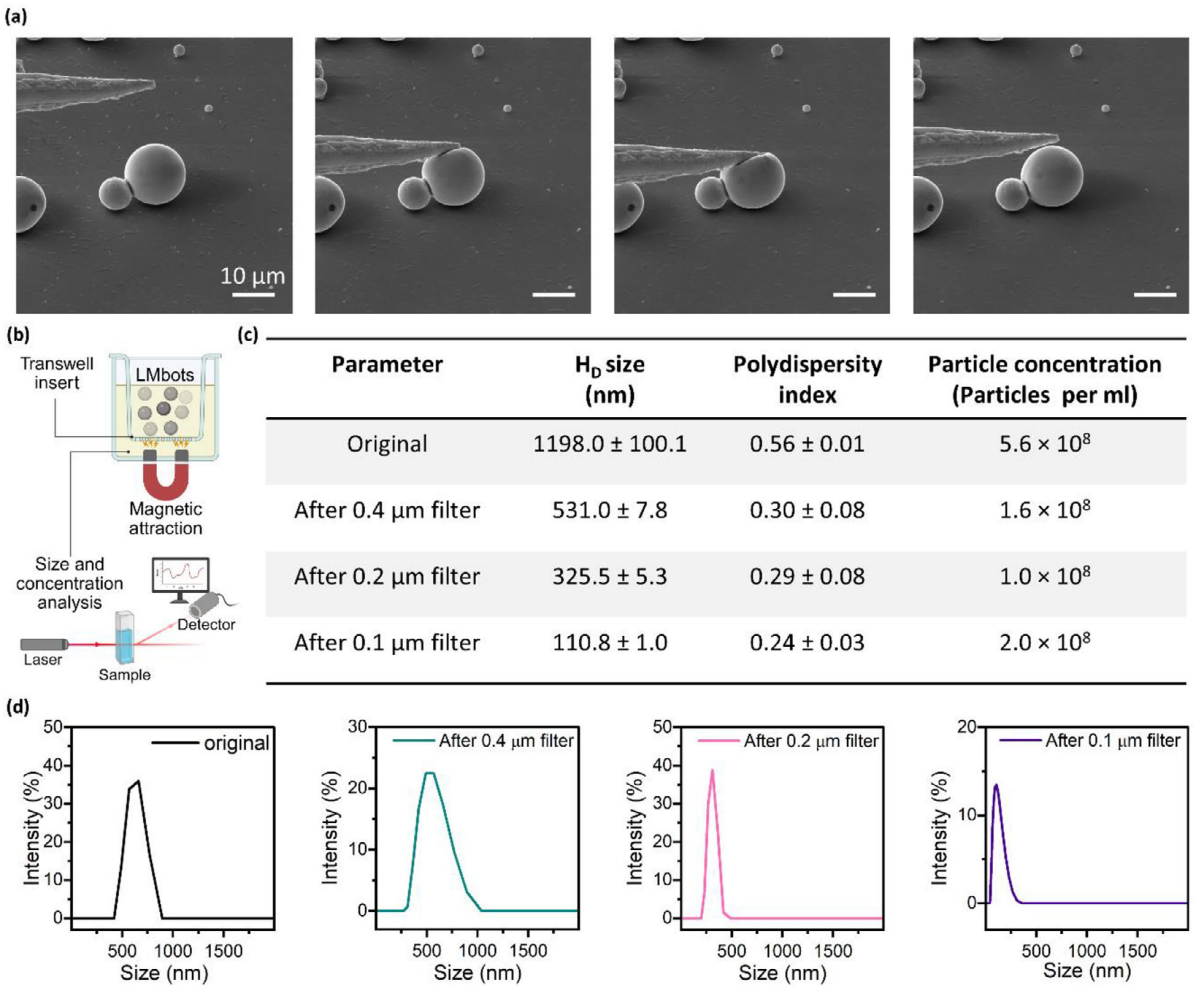
Deformation and traversing behavior of LMbots under static conditions. a) SEM images of individual LMbots showing shape deformation when mechanically pressed with a nanomanipulator tip. b) Schematic setup for analyzing the traversing behavior of LMbots using membrane filters with varying pore sizes. A permanent magnet was placed under the plate. After 30 min, LMbots that passed through the membranes were collected and analyzed by DLS. c) Summary of analyzed parameters (hydrodynamic diameter, polydispersity index, and particle concentration) of LMbots after traversing the membrane filter at sizes of 0.4, 0.2, and 0.1 µm. d) Hydrodynamic size distribution of LMbots after traversing the filters of different sizes.

As shown in **Figure** **6**a, the individual LMbots returned to their original shape after repeated deformation cycles when pressed by a nanomanipulator tip. Its overall structure remained intact even under substantial compression, with deformation ratios exceeding 80% without bursting. Video S6 (Supporting Information) compares the shape deformation of LMbots over more than five cycles under both mild and extensive pressing. Under both situations, the soft‐bodied LMbots exhibited excellent structural stability, elasticity, and mechanical resilience.

Another setup to test the deformation and traversing behavior was carried out using the filters with different membrane pore sizes, where the LMbots suspension was introduced into the upper chamber, and a permanent magnet was placed at the bottom of the setup (Figure 6b). Three types of membranes were used with filter pore sizes of 0.4, 0.2, and 0.1 µm, respectively. After half an hour, the aliquots from the bottom chamber were collected, and the aqueous solutions were analyzed by dynamic light scattering (DLS). The original dispersion of LMbots showed a size distribution of hydrodynamic diameter over 1 µm, where bigger NPs were beyond the detection range of DLS measurement (Figure 6c). After passing through a 0.4 µm filter, the number of collected LMbots reduced to less than 30% of the number of LMbots in the initial dispersion, with a significantly reduced hydrodynamic diameter of ≈500 nm. Similar phenomena were observed for 0.2 and 0.1 µm filters, where the sizes of LMbots were reduced to ≈300 and ≈100 nm, respectively. The reduced size closely matched the pore dimensions of the filter, and the filtered LMbots remained magnetically responsive. Moreover, the size distribution curves of the filtrates (Figure 6d) show a single peak without any multiple peaks, suggesting that the particles passing through the membrane exhibited a relatively uniform reduced size. When navigating through pores smaller than their original dimensions, the LMbots are likely subjected to strong mechanical stress and confinement‐induced breakage rather than active or reversible deformation. This process, therefore, represents partial fragmentation or disassembly rather than elastic shape adaptation as observed under direct mechanical pressing. Importantly, the resulting fragments still display magnetic responsiveness, indicating that the liquid metal matrix and embedded Fe cores remain functionally intact. These findings suggest that although the overall structural integrity of LMbots may be compromised under extreme confinement, their key magnetic and material functionalities can persist, enabling continued response to external magnetic fields.

### 2.6 Traversing Behavior of LMbots for Magnetically Guided Transvascular Navigation

Given the novelty of liquid metal‐based nanomaterials for biomedical applications, a comprehensive evaluation of these parameters, such as biodistribution, biodegradation, and potential organ accumulation, is essential before their deployment in biomedical contexts.^[^ ^35^ ^]^ A plethora of studies have demonstrated a generally favorable biocompatibility profile for gallium‐based liquid metal formulations. For instance, Wang and colleagues reported a Ga‐based liquid metals exhibit good hemocompatibility, as they do not induce hemolysis, have no significant impact on blood or serum components, and maintain stable surface properties after blood contact.^[^ ^36^ ^]^ Gu's group also illustrates both the functional versatility and favorable biosafety profile of gallium‐based nanomaterials in vivo.^[^ ^34^ ^]^ Collective results demonstrate that while rigorous biodistribution and long‐term toxicity studies remain imperative, current data support the endothelial safety and translational potential of liquid‐metal‐based microrobots.

**Figure 7:**
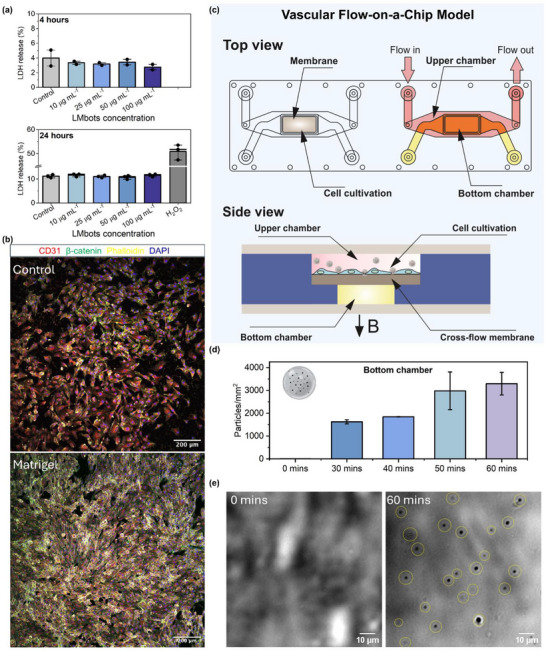
Transvascular navigation of LMbots under flow conditions in a vascular flow‐on‐a‐chip model. a) Cytotoxicity of LMbots was assessed by measuring LDH release from HUVEC cells following exposure to different doses of LMbots (10–100 µg mL^−1^) for 4 and 24 h, respectively. Cytotoxicity is expressed as a percentage relative to the maximum LDH release. The results represent the means ± SD of three independent determinations. Hydrogen peroxide (H_2_O_2_) treatment was used as a positive control to trigger cell death. Control represents untreated cells used to determine spontaneous LDH release. b) Confocal microscopy images of HUVECs cultured on control (uncoated) and Matrigel‐coated substrates for 48 h. Cells were stained for CD31 (red), β‐catenin (green), actin filaments (Phalloidin, yellow), and nuclei (DAPI, blue). Scale bar is 200 µm. c) Schematic illustration of the vascular flow‐on‐a‐chip model, with HUVEC cells growing on the upper membrane. The top view and side views are shown; the upper chamber is connected to the flow generated by a peristaltic pump. B (with arrows) indicates the direction of the applied magnetic field. d) Time‐dependent increase in LMbot numbers observed in the bottom chamber under flow conditions. e) Microscopic images of the bottom chamber at the start of flow and after 1 h of LMbots circulation, with yellow circles indicating the observed LMbots. Scale bar is 10 µm.

To corroborate these findings for LMbots, we assessed the cytotoxicity of LMbots for human umbilical vein endothelial cells (HUVECs) using a lactate dehydrogenase (LDH) release assay, which measures plasma membrane integrity as an indicator of cell viability. Across a wide concentration range (from 10 to 100 µg mL^−1^) of LMbots tested, LDH release from HUVECs remained comparable to that of untreated controls, indicating that LMbots exposure did not induce detectable cytotoxicity (**Figure** **7**a). To further strengthen the biocompatibility assessment, we additionally evaluated long‐term metabolic activity and intracellular oxidative stress. MTS assays performed after 48 and 72 h of continuous incubation, as well as endpoint and time‐resolved intracellular reactive oxygen species (ROS) measurements with 50 µg mL^−1^ of LMbots, showed no reduction in viability or increase in intracellular oxidative stress in LMbots‐treated cells (Figure S1, Supporting Information). In particular, the 120‐min kinetic ROS monitoring confirmed that ROS levels remained stable throughout the early exposure window, without any transient spikes or delayed‐onset oxidative responses. The close overlap between LMbots and control ROS traces throughout the entire time course further indicates that the presence of LMbots does not perturb basal redox homeostasis, even during dynamic intracellular ROS fluctuations. We note that 72 h represents the maximum practical culture duration without medium replacement, as media exchange would physically remove a substantial portion of LMbots and confound both cytotoxicity and potential ion‐leaching assessments. Within this uninterrupted window, LMbots maintained their structural integrity, and no ion‐related effects were observed, consistent with the known stability of GaIn alloys, which form a passivating oxide shell with minimal solubility under physiological conditions. These findings demonstrate that LMbots maintain endothelial cell viability under the tested conditions, supporting their biocompatibility for subsequent investigations of magnetically guided transvascular navigation.

Furthermore, to mimic physiological conditions and to evaluate the transvascular potential of LMbots, we aimed to establish a vascular flow‐on‐a‐chip microfluidic device. Such a microfluidic platform can recapitulate the dynamic shear stresses, endothelial barrier properties, and confined geometries of blood vessels, thereby providing a more relevant environment for studying LMbots–endothelium interactions. Before establishing a vascular flow‐on‐a‐chip system, it is essential to optimize endothelial cell adhesion and monolayer formation on the microfluidic device, as these parameters critically influence endothelial barrier integrity and reproducibility of the model.^[^ ^37^ ^]^ Substrate coatings are known to affect the extent of cellular adhesion, spreading, and junctional organization, which in turn dictate the physiological relevance of in vitro vascular assays.^[^ ^38^ ^]^

To identify the most suitable coating for HUVEC cells, Ibidi 18‐well glass‐bottom slides were functionalized with different extracellular matrix (ECM) proteins or their commercially available mixtures, including gelatin, collagen V, fibronectin, Cultrex, and Matrigel. HUVECs were seeded on the coated substrates and cultured for 48 h, after which adhesion and junctional integrity were evaluated by immunofluorescence staining for CD31 (an endothelial junction marker), β‐catenin (a component of adherent junctions), actin filaments, and nuclei, followed by confocal microscopy imaging. Among the tested substrates, Matrigel provided the most robust support for HUVEC adhesion and monolayer organization, as evidenced by strong and continuous CD31 staining along cell‐cell borders and well‐defined cytoskeletal structures (Figure 7b). These results highlight the critical influence of substrate selection on endothelial cell adhesion and underscore Matrigel as the most effective ECM coating for constructing physiologically relevant endothelial barriers in subsequent vascular flow‐on‐a‐chip assays. This superior performance is likely due to Matrigel's complex composition of laminin, collagen IV, entactin, and growth factors, which closely mimics the native basement membrane and promotes endothelial adhesion, spreading, and junction formation.^[^ ^37^ ^]^

To establish a vascular flow‐on‐a‐chip model, we employed a cross‐membrane microfluidic chip as the platform. This device was selected due to its cross‐membrane architecture, allowing independent control of apical and basal flow channels, enabling the formation of a well‐defined endothelial barrier and the simulation of physiological shear stresses across a thin, semipermeable membrane.^[^ ^39^ ^]^ To promote HUVEC adhesion and monolayer formation, the membrane was coated with Matrigel, which provides a basement‐membrane–mimetic extracellular matrix that supports endothelial spreading and junctional organization. HUVECs were then seeded onto the coated membrane and cultured under static conditions until reaching confluency, resulting in a continuous, functional endothelial monolayer suitable for subsequent studies of LMbots transvascular traversal under flow and magnetic guidance, where a magnetic force was applied downward toward the bottom chamber using a permanent magnet. (Figure 7c).

Following the establishment of the endothelial monolayer mimicking the blood vessel, we attached a peristaltic pump to the microfluidic channel, recreating physiological blood flow conditions within the vascular network. Investigating the transvascular behavior of LMbots is critical, as their ability to cross endothelial barriers under flow conditions is central to potential biomedical applications, including targeted drug delivery, minimally invasive vascular therapies, and in vivo diagnostic interventions. Dispersed LMbots were circulated through the upper chamber via the peristaltic pump at a controlled flow rate of ≈50 cm min^−1^, while a magnetic force was applied downward toward the bottom chamber of the vascular flow‐on‐a‐chip model. The translocation of LMbots through the endothelial barrier and membrane was monitored at multiple time points to quantitatively assess their kinetics and efficiency under physiologically relevant flow and shear conditions. As shown in Figure 7d, the number of transversed particles increased from zero to ≈1600 particles mm^−2^ in the first 30 min, doubling to ≈3200 particles mm^−2^ after 1 h of circulation. This accumulation pattern, also visualized in Figure 7e and Video S7 (Supporting Information), demonstrates that LMbots can effectively cross the endothelial barrier over time, highlighting their potential for sustained delivery across vascular networks.

### 2.7 Mechanistic Insights into LMbots Traversing and Relevance to Biological Systems

Understanding how LMbots interact with and cross biological barriers is essential for evaluating their biomedical potential. However, real‐time visualization of LMbots crossing biological barriers remains technically challenging due to tissue opacity, rapid motion, and imaging limits. To approximate this process under controllable conditions, we employed the porous cell‐free membrane‐based microfluidic platform that enables simultaneous magnetic guidance and optical monitoring. The LMbots were subjected to a permanent magnetic force from the bottom of the cross‐membrane chamber under no flow or flow conditions. As shown in Video S8 (Supporting Information), LMbots rolled along the membrane surface before gradually passing through pores of comparable or smaller size. During crossing, they experienced intermittent compression rather than continuous elastic deformation. Magnetically responsive LMbots appeared on the opposite side, confirming that both the Fe cores and liquid‐metal matrix remained intact. Size and morphology analysis (Figure S2, Supporting Information) revealed that flow broadened the particle‐size distribution and introduced smaller droplets (1 to 3 µm), consistent with confinement‐induced compression or breakage under combined magnetic and hydrodynamic forces. A subset of LMbots with sizes exceeding the pore diameter was observed to cross the membrane, especially under flow conditions. Furthermore, after crossing the membrane pores, particles larger than the pore size (8 µm) displayed a slightly elliptical morphology with intact surfaces, suggesting force‐induced deformation while maintaining overall structural integrity despite the loss of perfect sphericity (Figure S2e,g, Supporting Information). Despite these changes, the LMbots preserved metallic continuity and magnetic responsiveness, indicating that membrane passage occurs primarily via membrane permeation and mechanically assisted adaptation rather than magnetically driven large‐scale deformation.

For an 8 µm LMbot achieving ≈5% shape deformation, it requires overcoming its intrinsic capillary resistance. With an effective interfacial tension of ≈0.5 N m^−1^ for Ga‐In materials (although the value is much lower if considering the oxide layer as an elastic shell), the estimated pressure needed is 1 to 10 kPa, corresponding to a force of ≈50 to 500 nN. Under physiological conditions, this threshold can vary widely depending on the oxide layer integrity, the surrounding chemical environment, and confinement geometry. Factors such as local acidity, reductive species, or shear stress may weaken the oxide layer and lower the deformation barrier, while adhesion, wetting, and the presence of Fe inclusions influence mechanical response. Overall, the effective deformation threshold likely lies within 10 to 1000 nN under biologically relevant conditions, implying that subtle changes in interfacial chemistry or mechanical confinement can strongly modulate LMbot adaptability in vivo.

Theoretical estimation shows that under the applied magnetic field gradient (≈10^3^–10^4^ T m^−1^), the resulting magnetic stress on the liquid‐metal interface is only a few pascals, far below the pressure needed for visible deformation. Because the magnetic force depends on the field gradient (∇B) rather than absolute field strength, even strong fields produce limited mechanical stress if the gradient is shallow. In contrast, physiological blood flow can generate shear stresses of 1 to 10 Pa and dynamic pressures of 50 to 500 Pa, which, when combined with magnetic confinement, may locally reach hundreds of pascals. Such transient pressures, vortices, or wall interactions can induce compression or partial fragmentation, especially if oxide shell softening reduces interfacial tension of the LMbots. Indeed, a recent study similarly reported detailed optical evidence of flow‐assisted deformation of magnetic liquid‐metal droplets (coated with polymers) under combined hydrodynamic and magnetic forces, highlighting the cooperative role of these factors.^[^ ^17^ ^]^ Therefore, LMbots deformation and traversal are governed by the synergistic interplay of magnetic gradients and fluidic stresses, as well as the intrinsic surface chemistry, rather than by magnetic forces alone.

Although no significant magnetic‐induced deformation was observed during membrane crossing, the softness of the LMbots remains critical for biomedical performance. The liquid‐metal matrix provides mechanical compliance, allowing particles to withstand compression and shear without structural failure, unlike rigid or hydrogel‐based microrobots that often fragment under flow.^[^ ^40^, ^41^ ^]^ Even when partial fragmentation occurred, the droplets retained magnetic responsiveness, confirming preserved functionality. This compliant behavior supports safe contact and transient squeezing within confined geometries rather than large‐scale deformation. Liquid‐metal‐based microrobots are known to balance surface tension and deformability for durable, fluidic maneuverability.^[^ ^40^, ^41^ ^]^ Unlike earlier systems operating under static or low‐flow conditions,^[^ ^17^, ^21^, ^22^ ^]^ the present LMbots function reliably under physiological flow, exhibiting neutrophil‐like adaptability that facilitates migration across soft or leaky vascular barriers.^[^ ^42^, ^43^, ^44^ ^]^ Thus, even without active deformation, softness serves as a biophysical enabler, ensuring mechanical tolerance, flow adaptability, and biocompatible transvascular transport, advancing magnetically guided microrobots toward image‐guided therapy and targeted delivery.^[^ ^45^ ^]^

Looking ahead, LMbots can be further engineered for therapeutic integration through surface functionalization at the hydroxylated GaOOH/WO_x_ interface or by encapsulation within thin polymeric or porous shells (e.g., PLGA, PDA, mesoporous silica) to form LM@reservoir constructs. On‐demand release could be triggered by magneto‐mechanical stress during wall‐guided rolling, mild magnetothermal heating from the Fe‐doped core, or microenvironmental cues such as acidic pH or elevated ROS levels. These strategies align with previous liquid‐metal nanomedicine and surface‐chemistry approaches^[^ ^16^, ^34^, ^45^ ^]^ while leveraging the platform's intrinsic radiopacity and demonstrated biocompatible traversal. Future studies will quantify drug loading and controlled release under physiological flow using endothelial‐on‐chip and CAM tumor models, correlating delivery efficacy with real‐time fluoroscopic tracking.

## 3 Conclusion

The design of microrobots capable of navigating the bloodstream requires careful consideration of both mechanical properties and the dynamic vascular environment. Blood vessels present a complex landscape of varying diameters, branching networks, and pulsatile flow, which generates heterogeneous shear stresses that can impede conventional rigid microrobots. Magnetically guided microrobots with soft and deformable nature can dynamically adapt their shape in response to mechanical forces encountered during navigation, while magnetic fields provide steering rather than deformation. These microrobots also have the potential to reduce the risk of soft‐tissue damage. Research has shown that materials such as liquid metal,^[^ ^21^ ^]^ hydrogels,^[^ ^46^ ^]^ and flexible polymers^[^ ^47^ ^]^ enable the robots to dynamically change shape in response to external forces, allowing them to squeeze through narrow intercellular gaps, cross semipermeable membranes, and penetrate leaky or compromised vessel walls. Magnetic or acoustic actuation provides precise directional control, enabling the robots to swim against flow and adapt their propulsion strategies in real time, much like immune cells navigating toward sites of inflammation.^[^ ^17^, ^27^ ^]^

Compared with previously reported liquid‐metal‐based microrobots, which primarily demonstrated shape reconfiguration or droplet manipulation in static or low‐flow conditions,^[^ ^17^, ^21^, ^22^ ^]^ the LMbots developed in this study uniquely combine three key advantages: ability to cross narrow or obstructed vessels, intrinsic radiopacity enabling real‐time fluoroscopic tracking,^[^ ^4^ ^]^ and endothelial biocompatibility validated under physiological flow in a vascular flow‐on‐a‐chip model. These integrated features bridge the gap between proof‐of‐concept soft liquid‐metal microrobots and clinically relevant transvascular navigation, providing a versatile platform for image‐guided therapy and targeted delivery. Table S1 (Supporting Information) summarizes the actuation mode, typical locomotion speed, control precision, deformability mechanism, and imaging capability reported for selected prior works and for the present soft and liquid metal‐based microrobots. Unlike previous systems, the LMbots reported in this work combine controllable rolling motion, mechanical deformability (rather than direct magnetic deformation), and intrinsic radiopacity, enabling real‐time X‐ray–guided navigation under physiological flow conditions. Their unique ability to deform while sustaining functional payloads positions them as a versatile platform for targeted therapeutic delivery, real‐time in vivo tracking, and minimally invasive interventions. This combination of softness and active guidance allows LMbots to maintain position, avoid clearance, and reach target sites even under high flow conditions. Furthermore, their ability to interact mechanically with the endothelium introduces opportunities for transvascular transport, targeted therapeutic delivery, and minimally invasive interventions, creating a bridge between micro‐scale robotics and physiological function. Such designs not only replicate aspects of natural cellular behavior but also expand the capabilities of microscale machines in clinical and research applications.

Recently, Landers et al.^[^ ^48^ ^]^ demonstrated the feasibility of magnetically guided therapeutic microdevices in large‐animal models under clinically realistic magnetic field strengths, further underscoring the translational relevance of magnetic actuation regimes comparable to those used in our study. From a translational perspective, the magnetic field strength used in this study in the tens of mT range falls well within the range achievable by clinically available electromagnetic steering systems and MRI‐based magnetic navigation setups supporting potential adaptation to real‐time vascular procedures. The fluoroscopic imaging energies employed here are likewise consistent with standard interventional radiology practices, ensuring clinical compatibility for image‐guided control. In terms of scalability, the liquid‐metal dispersion process can be adapted to sonication‐based fabrication for uniform, large‐volume production. The GaIn/Fe composite and surface layer exhibit high chemical and oxidative stability, allowing ethanol‐ or gamma‐sterilization and extended storage without degradation. These characteristics, together with future optimization of polymer‐encapsulated LM@Fe architectures, form the basis for advancing LMbots toward industrial production and pre‐clinical evaluation.

## 4 Experimental Section

### Materials

Fe nanoparticles (99.9%, 100 to 300 nm) were purchased from Aladdin Reagent Co., Ltd (Shanghai, China). Other chemicals, including gallium (99.999%), indium (99.999%), hydrochloric acid (37%), sodium tungstate dihydrate, and ethanol (absolute), were purchased from Sigma–Aldrich (Germany) unless mentioned otherwise.

### LMbots Fabrication

The fabrication of GaIn‐Fe‐based LMbots followed our previous protocols with slight modifications.^[^ ^17^, ^22^ ^]^ Ga and In were mixed in a weight ratio of 3:1. Fe nanoparticles in the weight ratio of 1/10 of GaIn alloy were mixed with the alloy and 5 m HCl. The mixture was vigorously shaken for 10 min or until Fe was fully incorporated into the GaIn alloy. The liquid metal mixture was extracted using a transfer pipette and added to a 0.1 m Na_2_WO_4_ solution adjusted to pH 3 by HCl. The mixture was sonicated using a tip sonicator at 100 W for 10 min in an ice bath, followed by three washes with ethanol and deionized water. A permanent magnet was used during washing to retain the synthesized LMbots.

### LMbots Physicochemical Characterization

Morphological and compositional characterization were carried out with a MIRA 3 XMU scanning electron microscopy (SEM) equipped with an Oxford energy dispersive X‐ray (EDX) detector (Tescan, Czech Republic). The SEM images were obtained at 5 kV with a secondary electron detector. X‐ray diffraction (XRD characterization was carried out using a SmartLab 3 KW diffractometer equipped with a Cu K*α* anode X‐ray tube, operated at 40 KV and 30 mA (Rigaku, Japan). Crystallite size calculations were carried out based on XRD data using the *Scherrer* equation. Surface chemical composition of LMbots was characterized by X‐ray photoelectron spectroscopy (XPS) using an Axis Supra instrument with monochromatic Al K*α* (1486.7 eV) source (Kratos Analytical, UK). All spectra were calibrated according to the adventitious C 1s peak at 284.8 eV and fitted using KolXPD software (kolibrik.net). Hydrodynamic diameter, zeta potential, and nanoparticle concentrations were measured in water with dynamic light scattering (DLS) Panalytical Zetasizer Ultra (Malvern Panalytical, UK). A vibrating sample magnetometer (VersaLab VSM, Quantum Design, USA) was used to measure the saturation magnetization of samples at 300 K with an applied magnetic field ranging from ‐15 to 15 kOe.

### Magnetic Navigation of LMbots

Magnetic motion of LMbots was recorded using an ECLIPSE TS2R microscope and a BASLER acA1920‐155uc camera (Nikon, Japan). A customized magnetic setup with three orthogonal coil pairs on a home‐built support generated a transverse rotating magnetic field. The microrobots were controlled under 3 mT magnetic fields at frequencies from 0 to 100 Hz, and the videos were analyzed with NIS‐Elements Advanced Research software and ImageJ software. Two types of microfluidic channels were used: the straight channels were purchased from Ibidi µ‐Slide VI 0.1 (uncoated). The curved and branched channels were purchased from Microfluidic ChipShop (Germany), droplet generator chip Fluidic 285. The flow was generated by an external Infuse/Withdraw Pump 11 Pico Plus Elite (Harvard Apparatus, USA) connected through syringes.

### LMbots Behavior Tested on *ex ovo* Chorioallantoic Membrane Model

*Ex ovo* quail CAM assay was performed according to Petrovova et al.^[^ ^49^ ^]^ Briefly, the fertilized quail (*Coturnix japonica*) eggs were obtained from a quail farm at the Department of Animal Breeding at Mendel University in Brno (Brno, Czech Republic). The eggs were incubated horizontally at 37.5 °C and 65% relative humidity. At 2.5 days post‐incubation, the eggshells were disinfected with 70% ethanol, and the contents were carefully transferred into sterile 6‐well cell culture plates. The embryos were then incubated in a still‐air incubator under the same temperature and humidity conditions until embryonic day 11 (ED11). At this stage, CAM and embryo were utilized to investigate the movement of LMbots following subcutaneous and intravenous injections, with their distribution navigated by an externally applied magnetic field. For experiments involving xenografted prostate tumors, ≈5×10^5^ 22Rv1 human prostate cancer cells were carefully placed onto the CAM surface on embryonic day 6 (ED6). The embryos were further incubated under the same conditions until ED11. At this point, the CAM was analyzed for the distribution and movement of LMbots in the context of targeting the human prostate tumor implanted on the CAM. In EU countries, the CAM assay does not require ethical approval.

### Radiographic Analysis of LMbots Using X‐ray Fluoroscopy

The radiopacity of LMbots was evaluated with the OEC 9800 PLUS C‐arm (GE OEC Medical Systems, Inc., USA). Commercial contrast agent Ultravist^®^ 300 (Iopromidum, Bayer, USA) containing 300 mg mL^−1^ of iodine was used as a control. Fresh porcine hearts were obtained from a local butchery and used within 24 h of harvesting, and transported on ice. Porcine hearts were obtained from a licensed local abattoir as byproducts of animals slaughtered for food production. No animals were sacrificed specifically for this study; therefore, ethical approval was not required. Images were acquired using an exposure setting of 1 mA and 48 kV under ex vivo tissue‐free conditions, and 1–3 mA and 55 kV when imaging through tissue. Images were analyzed via ImageJ software and MicroDicom software.

### Shape Deformation of LMbots

The shape deformation of LMbots was carried out using a focused ion beam‐scanning electron microscope (FIB‐SEM, Tescan LYRA3, Czech Republic) equipped with two nanomanipulators. Samples dispersed in ethanol were drop‐cast onto a silicon wafer and mounted on the sample stage of the FIB‐SEM. The stage was tilted at 55°, and a nanomanipulator equipped with a tungsten probe tip ≈2 µm in diameter at its thinnest part was used to probe individual LMbots. SEM images were taken with an accelerating voltage of 20 KV. For the traversing experiments under static conditions, filters of PVDF membranes with pore sizes of 0.1, 0.2, and 0.4 µm (Sigma–Aldrich) were used. 0.5 mL of LMbots suspensions was added into the upper part, and cube‐shaped (2 × 2 × 2 cm) NdFeB permanent magnets were placed under the setup for 30 min. After 30 min, the aliquots passed through the filter were collected and analyzed by DLS with Zetasizer Ultra (Malven Panalytical, UK).

### Establishment of a Vascular Flow‐on‐a‐Chip Model

Human Umbilical Vein Endothelial Cells (HUVECs) were obtained from the American Type Culture Collection (ATCC, USA, CRL‐4053; HUVE/TERT2, an hTERT‐immortalized endothelial cell line). Cells were cultured in Human Endothelial Serum‐Free Medium (HE‐SFM; Gibco, #11 111 044) supplemented with 10% fetal bovine serum (FBS, Biowest # BWSTS1400‐500), human fibroblast growth factor‐B (hFGF‐B, STEMCEL # 78 003, 20 ng mL^−1^), human epidermal growth factor (hEGF, Bio‐techne # 236‐EG, 10 ng mL^−1^), bovine fibronectin (Sigma–Aldrich # F1141‐5MG, 10 µg mL^−1^), and 1% penicillin/streptomycin (Capricorn Scientific #PS‐B). Cells were maintained at 37 °C in a humidified atmosphere containing 5% CO_2_.

Cytotoxicity of LMbots was evaluated using the CyQUANT™ LDH Cytotoxicity Assay Kit (Cat. No. C20301, Invitrogen, USA) was used to determine the cytotoxic effect of LMbots in HUVEC cells. HUVEC cells were incubated with different concentrations (10‐100 µg mL^−1^) of LMbots for 4 and 24 h. To determine maximum LDH activity, the lysis buffer was added to HUVEC cells, and the plate was then incubated for 45 min at 37 °C in 5% CO_2_. Hydrogen peroxide (H_2_O_2_) treatment was used as a positive control to induce oxidative stress and cell damage. Untreated cells were used as a control to determine spontaneous LDH activity. To perform the assay, an aliquot of the cell culture media was transferred to a new 96‐well plate, and the CyQUANT™ LDH Cytotoxicity Assay Kit reaction mixture was added. After a 30‐min incubation at room temperature, the assay was stopped by adding the stop solution, and then absorbance was measured using a microplate reader (Multiscan GO, Thermo Scientific) at 490  and 680 nm. The LDH activity was calculated by subtracting the 680‐nm absorbance (background) value from the 490‐nm absorbance. Then, cytotoxicity was calculated using the following formula: Cytotoxicity (%) = ((Experimental LDH ‐ Medium Background)/(Maximum LDH ‐ Medium Background)) × 100.

For long term cytotoxicity MTS assay, HUVECs were seeded at a density 10 000 cells/well in a 96‐well cell culture plate and incubated overnight in a humidified atmosphere containing 5% CO_2_ at 37 °C. Subsequently, HUVEC were treated with LMbots at a final concentration 50 µg mL^−1^ and incubated for an additional 48 or 72 h. As a positive control, 5% of dimethyl sulfoxide (DMSO) was added to HUVECs 1 h before the end of incubation time to induce cell death. No media was changed during the whole incubation time. After incubation, the cell viability was assessed using the MTS assay (Promega, #G3582) according to the manufacturer's instructions. Absorbance was measured on Multiscan GO (Thermo Scientific) at 490 nm. A reference wavelength of 630 nm was used to subtract the background. The average 490 nm absorbance from the “no cell” control wells was subtracted from all other absorbance values to yield corrected absorbances.

For intracellular ROS detection, HUVECs were seeded at a density of 10 000 cells/well in 96‐well black/clear bottom cell culture and incubated overnight in a humidified atmosphere containing 5% CO_2_ at 37 °C. Subsequently, HUVEC were treated with LMbots at a final concentration 50 µg mL^−1^. As positive controls for ROS induction 200 µm H_2_O_2_ or 100 µm of tert‐Butyl hydroperoxide (TBHP) was added to HUVECs 1 h before the end of incubation time. No media was changed during the whole incubation time. The intracellular ROS detection kit (Sigma–Aldrich, #MAK143) was used according to the manufacturer's recommendations. Short‐term intracellular ROS induction by LMbots was monitored kinetically at 10‐min intervals for a total of 120 min at 37 °C. Long‐term ROS levels were subsequently assessed as by end‐point measurement after 48 and 72 h. Data were acquired on a Biotek FLx800 fluorescence microplate reader using an excitation filter 485/20 nm and an emission filter 528/20 nm.

To optimize the effect of substrate coating promoting cell adhesion, Ibidi 18‐well glass‐bottom slides (#81 817, Ibidi) were coated with either 0.1% gelatin (in water, Sigma–Aldrich # G1393), collagen I (IBIDI # 50 201, 5 µg cm^−2^ in 0.1 m acetic acid), fibronectin (Sigma–Aldrich # F1141‐5MG, 5 µg cm^−2^ in phosphate buffered saline, PBS), Cultrex (R&D systems # #: 3533‐010‐02, diluted 100× in PBS), or Matrigel (Corning # 356 231, diluted 100× in PBS). A volume of 50 µL coating solution was added per well, and slides were incubated for 60 min at 37 °C. Following incubation, excess solution was removed; protein‐coated wells (gelatin, collagen, fibronectin) were rinsed once with PBS, whereas Matrigel‐ and Cultrex‐coated wells were used directly without washing. HUVEC cells were seeded at a density of 1.5 × 10^5^ cells per well. Cells were cultured for 48 h at 37 °C and 5% CO_2_. Cells were fixed with 4% paraformaldehyde (PFA) for 20 min at 4 °C, washed three times with PBS, and permeabilized with 0.2% Triton X‐100 (Sigma–Aldrich # T8787) in PBS for 3 min. After blocking in 2.5% bovine serum albumin (BSA, Biowest #P6154‐KG, diluted in PBS) for 60 min, samples were incubated with primary antibodies against β‐catenin (rabbit, 1:200, Cell Signaling Technology #8480S) and CD31 (mouse, 1:200, Abcam #24 590) diluted in 0.5% BSA in PBS overnight at 4 °C. Following three PBS washes, cells were incubated with Alexa Fluor 488 goat anti‐rabbit IgG (1:500, Invitrogen #A11034), Alexa Fluor 546 goat anti‐mouse IgG (1:500, Invitrogen #A11030), and Phalloidin‐Atto647 (Sigma–Aldrich # 65 906, 1:500) diluted in 0.5% BSA in PBS for 1 h at room temperature in the dark. Following three PBS washes, the nuclei were counterstained with DAPI (Roche # 10 236 276 001, 4′,6‐diamidino‐2‐phenylindole, diluted 16000× in PBS) for 5 min at room temperature in the dark. After a final PBS wash, slides were imaged using confocal laser scanning microscopy (Zeiss LSM 780 confocal station, Germany).

To construct the vascular flow‐on‐a‐chip model, fluidic 480 microfluidic cross‐membrane chips with hydrophilic coatings and a pore size ≈ 8 µm were purchased from Microfluidic ChipShop (Germany). The membranes of the chips were coated with Matrigel for 1 h from the upper chamber. HUVEC cells were seeded at a density of 1.5 × 10^5^ cells cm^−2^ and cultured for 144 h at 37 °C and 5% CO_2_. Medium was changed daily. To establish the vascular flow‐on‐a‐chip model, chips were perfused with medium or medium containing LMbots using a peristaltic pump (Harvard Apparatus) at a flow rate of ≈50 cm min^−1^ (0.1 mL min^−1^ with a tube with an inner diameter of 0.5 mm). For measuring LMbots crossing the chip, a 2 × 2 × 2 cm NdFeB permanent magnet was placed underneath the chip membrane during the perfusion. The bottom chamber of the chip was observed via a 40× objective by an inverted optical microscope (Nikon, Japan). The quantification of LMbots was counted, and images were processed via ImageJ software.

### Statistics

All data were pre‐processed by background subtraction and normalization according to the corresponding assay requirements (e.g., LDH corrected for 680 nm background, MTS corrected for no‐cell controls, ROS fluorescence corrected for blank wells). Outliers were evaluated using the Grubbs test and removed only when statistically justified. Quantitative results were presented as mean ± standard deviation (SD). Unless otherwise stated, all biological experiments were performed with three independent biological replicates. Comparisons between two groups were assessed using a two‐tailed unpaired Student's t‐test, whereas analyses involving more than two groups were evaluated using one‐way ANOVA followed by Tukey's post‐hoc test to adjust for multiple comparisons. Statistical significance was defined as α = 0.05, with exact P‐values reported in the text or figure captions. All analyses were performed using OriginPro 2025, and significance levels are indicated as ^*^p < 0.05, ^**^p < 0.01, ^***^p < 0.001.

## Conflict of Interest

The authors declare no conflict of interest

## Author Contributions

X. J. conceived the concept, designed and carried out the experiments, and drafted/revised the manuscript. R. V. designed and performed the in vitro microfluidic chip experiments. X.W. performed the fabrication of LMbots. M. A. M. R. and Z. H. performed the CAM experiments. K. B. and J. F. performed the in vitro LDH and coating experiments. M.P. supervised the project and acquired funding. All authors have approved the final version of the manuscript.

## Supporting information



Supporting Information

Supplemental Video 1

Supplemental Video 2

Supplemental Video 3

Supplemental Video 4

Supplemental Video 5

Supplemental Video 6

Supplemental Video 7

Supplemental Video 8

## Data Availability

The data that support the findings of this study are available from the corresponding author upon reasonable request.
