# Supplementary material for: Liquid Metal Microrobots for Magnetically Guided Transvascular Navigation
Source: Adv Mater. 2025 Dec 19;38(20):e18382. doi: 10.1002/adma.202518382 (PMC13054206; doi:10.1002/adma.202518382)
Supplement: Supplementary file 1 — Supporting Information [file ADMA-38-e18382-s004.docx]

Supporting Information

Liquid Metal Microrobots for Magnetically Guided Transvascular Navigation

Xiaohui Ju, Roshan Velluvakandy, Xianghua Wu, Miguel Angel Merlos Rodrigo, Zbyněk Heger, Kamila Bendíčková, Jan Frič, Martin Pumera*

Note S1: Critical speed calculation and near-wall flow for LMbots

We derive a compact criterion for when a 10 µm liquid-metal microrobot (LMbot) can roll against a laminar flow near a wall, combining (i) a magnetic-friction--limited critical speed, and (ii) the reduced local fluid velocity in the near-wall region (Poiseuille no-slip).

1. **Critical speed (magnetic *vs.* viscous balance)**

A simple wall-rolling model equates the tangential viscous drag with the available magnetic-adhesive force:

$$U_{c}=\left( \mu_{k}m\nabla B \right)/6\pi\mu a$$

$$m=V\chi B/\mu_{0}=\left( 4\pi a^{3}\chi B \right)/\left( 3\mu_{0} \right)$$

Where *U_c_* is the critical rolling velocity (threshold where adhesion and rolling becomes unstable), $\mu_{k}$ is the effective friction coefficient between the LMbots and the wall, *m* is the magnetic moment, $\nabla B$ is the magnetic field gradient, $\mu$ is the dynamic viscosity of the surrounding fluid, *a* is the LMbot radius, *B* is the magnetic field strength and $\mu_{0}$ is the magnetic permeability of vacuum. For fixed $B$ and $\chi$, $U_{c}\propto a^{2}\nabla B$. Assume a 10 µm particle at 3 mT in water, the above equation to express *U_c_* can be summarized as:

$$U_{c}\approx0.0133\mu_{k}\chi\left( \nabla B \right)$$

1. **Near-wall flow (Poiseuille no-slip)**

In a laminar channel flow, the local speed near the wall is approximately linear:

$$\text{u}\left( \text{y} \right)\text{≈}\left( \text{du/dy} \right)_{\text{w}}\text{y=}\left( \text{4}\text{U}_{\text{avg}}\text{/}\text{R} \right)\text{y}$$

where $\text{u}\left( \text{y} \right)$ is the local flow velocity at distance *y* from the wall, $\left( \text{du/dy} \right)_{\text{w}}$ is the velocity gradient at the wall, $\text{U}_{\text{avg}}$ is the cross-sectional average flow speed, R is the channel radius, and y is the distance from the wall. For a spherical LMbot touching the wall, its center lies at *y=a*, so the local flow speed at the LMbot is:

$$\text{u}\left( \text{a} \right)\text{=(4}\text{U}_{\text{avg}}\text{a)}\text{/}\text{R}$$

With U_avg_ at 10 mm s^-1^, R at 0.5 mm, a at 5 µm, the low flow velocity near the wall is approximately 0,4 mm s^-1^. At a bulk flow of 10 mm s^-1^, the local near-wall speed is ~25 times slower due to the no-slip boundary.

1. **Combined feasibility of criteria for LMbot rolling against the flow**

Rolling against the flow is possible if the magnetic adhesive capacity exceeds the local viscous drag:

$$Uc\geq u\left( a \right)$$

$$\mu_{k}m\nabla B/6\pi\mu a\geq4U_{avg}a/R$$

Substituting the simplified form gives:

$${\nabla B}_{min}\geq0.40/\left( 0.0133\mu_{k}\chi\right)$$

Thus, at conservative conditions where $\chi$ is 1, $\mu_{k}$ equals to 0.3, with ${\nabla B}_{min}$ estimated to be ~100 T m^-1^, the calculated U_c_ critical speed is around 0,4 mm s^-1^. Under these conditions, a 10 µm LMbot at 3 mT can roll against the near-wall flow. In contrast, against-flow motion is not possible in the centerline region, where the flow velocity is around 10 mm s^-1^.


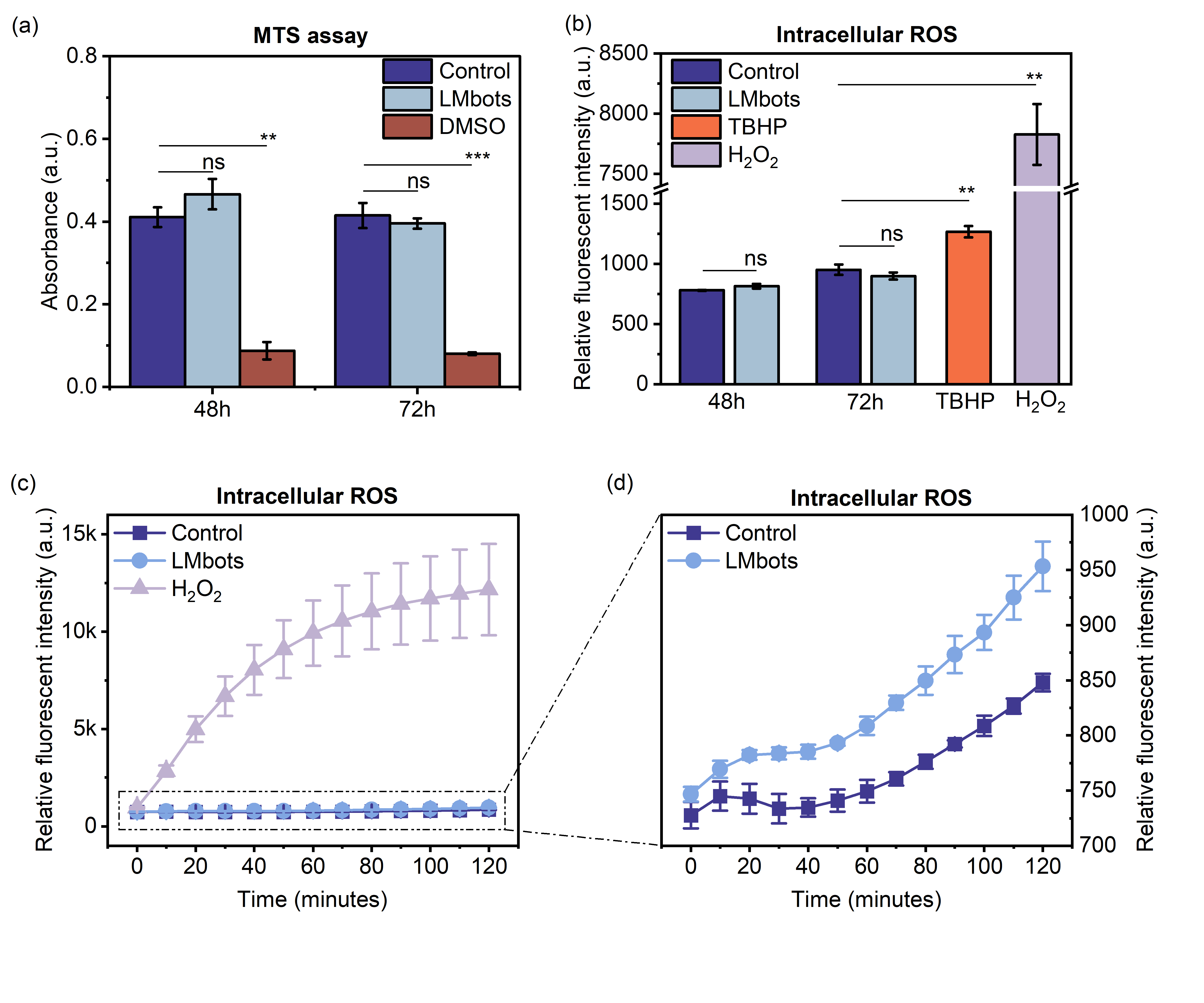


**Figure S1. Cytotoxicity and intracellular ROS analysis of LMbots in HUVEC cells. (a)** Long term cell cytotoxicity assessment via MTS assay shows cell metabolic activity after 48 h and 72 h incubation with LMbots. 5% of dimethyl sulfoxide (DMSO) was used as control to induce cell cytotoxicity. **(b)** Endpoint intracellular ROS measurement via an intracellular ROS assay kit to quantify cellular oxidative status in control and LMbots groups, with 100 µM of TBHP or 200 µM of H_2_O_2_ included as oxidative stress-inducing positive controls. **(c)** Time-resolved intracellular ROS kinetics recorded over short time range (within 120 minutes) to monitor dynamic changes in cellular oxidative state following treatment with LMbots, with 200 µM of H_2_O_2_ included as a positive oxidative-stress control. **(d)** Magnified view of the low-intensity ROS kinetic region to visualize baseline ROS dynamics in control and LMbots groups. Data were presented as mean ± standard deviation from three independent replicates. Statistical significance was determined by a two-tailed unpaired Student’s t-test (Welch’s correction applied when variances were unequal).; ns = not significant, *p < 0.05, **p < 0.01, ***p < 0.001.

**
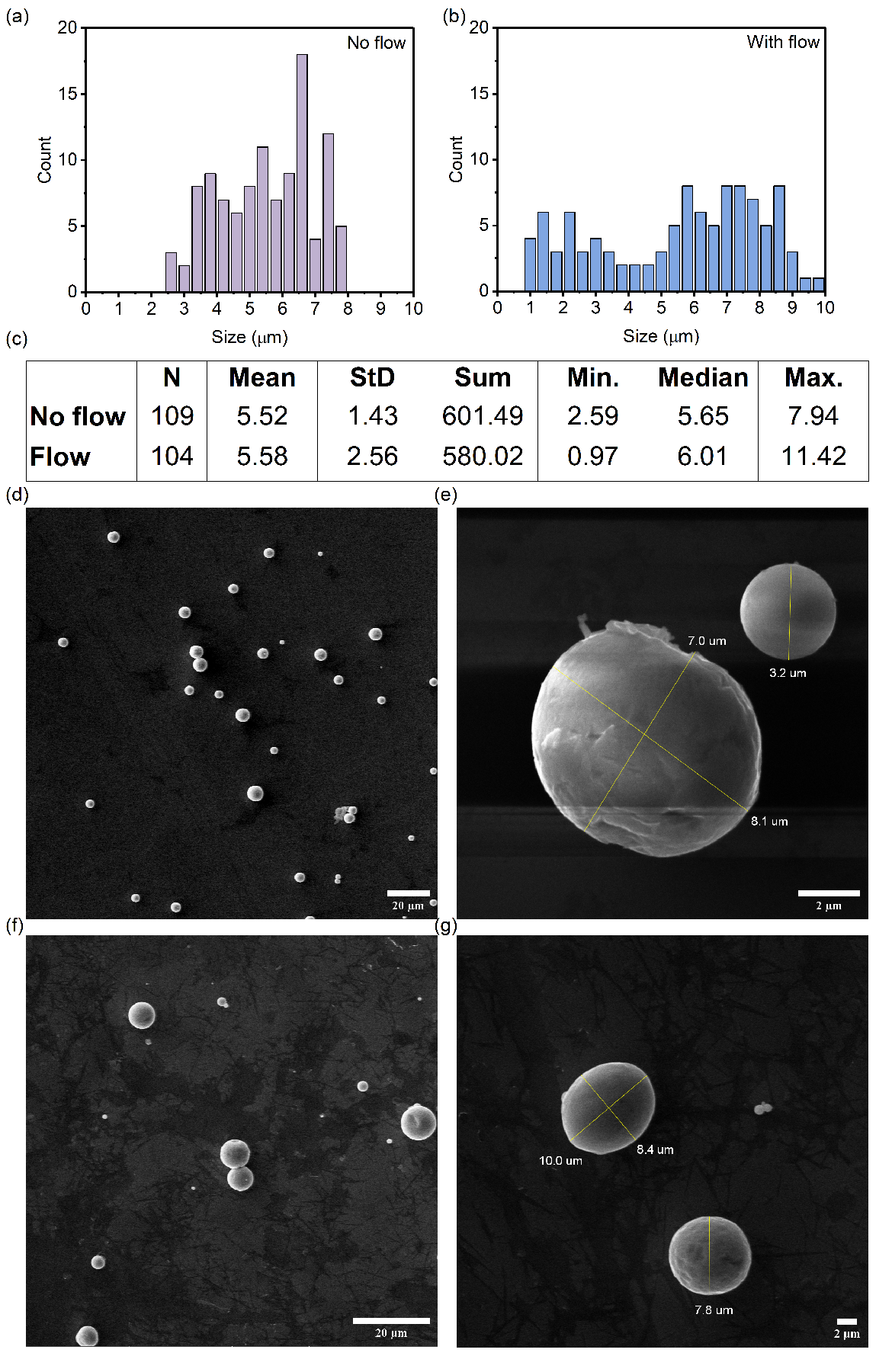
Figure S2. Size analysis of LMbots after crossing an 8 µm-pore membrane under a permanent magnetic field. (a)** Size distribution of LMbots after crossing without flow. **(b)** Size distribution after crossing under a flow rate of 50 cm min^-1^. **(c)** Statistical comparison of LMbots size distributions after membrane crossing. **(d–e)** Morphological characterization of LMbots after membrane crossing under no-flow conditions. **(f–g)** Morphological characterization of LMbots after membrane crossing under flow conditions.

**Table S1. Comparison of representative soft or liquid-metal microrobots and related systems.**

| **System / Reference** | **Actuation Mode** | **Typical Speed** | **Control Precision** | **Deformability** | **Imaging / Functional Capability** |
| --- | --- | --- | --- | --- | --- |
| **Millimeter-scale soft robots with polymer matrix^1^** | Magnetic + elastic body deformation | Not specified | High | Soft-bodied bending and multimodal shape change | Optical |
| **Ferromagnetic liquid robot^2^** | Permanent magnet | Not specified | Low | Stiffness-tunable ferromagnetic liquid droplet | Optical |
| **Magnetic lipiodol micro-droplets^3^** | External rotating magnetic field | ~ 6 µm/s | Moderate | Adaptive deformation | X-ray fluoroscopy + optical |
| **Magnetic liquid metal microrobots^4-6^** | External gradient and rotating fields | ~ 4-6 µm/s | High | Self-adaptive deformation crossing barriers | Optical |
| **Pt-coated liquid metal microrobots^7^** | Self-phoretic catalytic propulsion | ~10 µm/s | Low | Local interfacial flow deformation | Optical |
| **This work (LMbots)** | External gradient and rotating fields | ~50 µm/s | High | Mechanical deformation; no direct magnetic deformation | X-ray fluoroscopy + optical |

**REFERENCE**

(1) Hu, W.; Lum, G. Z.; Mastrangeli, M.; Sitti, M. Small-Scale Soft-Bodied Robot with Multimodal Locomotion. *Nature* **2018**, *554* (7690), 81-85.

(2) Li, Z. F.; Zhang, S. F.; Wang, Q.; Xu, Y. Z.; Li, Y. K.; Chen, X. J.; Chen, P.; Chen, D. Z.; Shi, Y. S.; Su, B. Untethered & Stiffness-Tunable Ferromagnetic Liquid Robots for Cleaning Thrombus in Complex Blood Vessels. *Advanced Materials* **2024**, *36* (46), 2409142.

(3) Ren, E.; Hu, J.; Mei, Z. Y.; Lin, L.; Zhang, Q.; He, P.; Wang, J. Q.; Sheng, T.; Chen, H.; Cheng, H. W.; et al. Water-Stable Magnetic Lipiodol Micro-Droplets as a Miniaturized Robotic Tool for Drug Delivery. *Advanced Materials* **2025**, *37* (3), 2412187.

(4) Wu, X.; Peng, X.; Ren, L.; Guan, J.; Pumera, M. Reconfigurable Self-Assembling Photocatalytic Magnetic Liquid Metal Microrobot Swarm for Microplastic Capture and Degradation. *Small* **2025**, 2501351.

(5) Wu, X. H.; Peng, X.; Ren, L.; Guan, J. G.; Pumera, M. Reconfigurable Magnetic Liquid Metal Microrobots: A Regenerable Solution for the Capture and Removal of Micro/Nanoplastics. *Advanced Functional Materials* **2024**, *34* (51), 202410167.

(6) Wu, X. H.; Zhang, L.; Tong, Y. F.; Ren, L.; Guo, H. R.; Miao, Y.; Xu, X.; Ji, Y.; Mou, F. Z.; Cheng, Y.; et al. Self-Adaptive Magnetic Liquid Metal Microrobots Capable of Crossing Biological Barriers and Wireless Neuromodulation. *ACS Nano* **2024**, *18* (43), 29558-29571.

(7) Wang, Y.; Duan, W.; Zhou, C.; Liu, Q.; Gu, J.; Ye, H.; Li, M.; Wang, W.; Ma, X. Phoretic Liquid Metal Micro/Nanomotors as Intelligent Filler for Targeted Microwelding. *Advanced Materials* **2019**, *31* (51), 1905067.
